# Chapparvovirus DNA Found in 4% of Dogs with Diarrhea

**DOI:** 10.3390/v11050398

**Published:** 2019-04-27

**Authors:** Elizabeth Fahsbender, Eda Altan, M. Alexis Seguin, Pauline Young, Marko Estrada, Christian Leutenegger, Eric Delwart

**Affiliations:** 1 Vitalant Research Institute, San Francisco, CA 94118, USA; efahsbender@vitalant.org (E.F.); EAltan@vitalant.org (E.A.); 2 Dept. of Laboratory Medicine, University of California, San Francisco, CA 94118, USA; 3 IDEXX Reference Laboratories, -Inc., West Sacramento, CA 95605, USA; alexis-seguin@idexx.com (M.A.S.); Pauline-Young@idexx.com (P.Y.); Marko-Estrada@idexx.com (M.E.); Christian-Leutenegger@idexx.com (C.L.)

**Keywords:** parvovirus, viral metagenomics, canine chapparvovirus

## Abstract

Feces from dogs in an unexplained outbreak of diarrhea were analyzed by viral metagenomics revealing the genome of a novel parvovirus. The parvovirus was named cachavirus and was classified within the proposed *Chapparvovirus* genus. Using PCR, cachavirus DNA was detected in two of nine tested dogs from that outbreak. In order to begin to elucidate the clinical impact of this virus, 2,053 canine fecal samples were screened using real-time PCR. Stool samples from 203 healthy dogs were positive for cachavirus DNA at a rate of 1.47%, while 802 diarrhea samples collected in 2017 and 964 samples collected in 2018 were positive at rates of 4.0% and 4.66% frequencies, respectively (healthy versus 2017-2018 combined diarrhea *p*-value of 0.05). None of 83 bloody diarrhea samples tested positive. Viral loads were generally low with average real-time PCR Ct values of 36 in all three positive groups. The species tropism and pathogenicity of cachavirus, the first chapparvovirus reported in feces of a placental carnivore, remains to be fully determined.

## 1. Introduction

Canine diarrhea is one of the most common illnesses treated by veterinarians with many possible causes of canine diarrhea, including bacteria, parasites, and viruses [1]. One of the most important dog enteric viruses is canine parvovirus 2 (CPV-2) in the *Carnivore protoparvovirus species 1* [2]. Parvoviruses are small, icosahedral, nonenveloped, single-stranded DNA viruses that are pathogenic to a variety of mammals [3,4,5]. The vertebrate-infecting parvoviruses are classified in the subfamily *Parvovirinae* in the *Parvoviridae* family (which also includes the insect infecting subfamily *Densovirinae*). The *Parvovirinae* subfamily is currently subdivided into eight officially recognized genera (*Dependoparvovirus*, *Copiparvovirus*, *Bocaparvovirus*, *Amdoparvovirus*, *Aveparvovirus*, *Protoparvovirus*, *Tetraparvovirus*, and *Erythroparvovirus* [6]). The recently proposed genus *Chapparvovirus* is currently comprised of a rat parvovirus 2 (KX272741) [7], *Eidolon helvum* fruit bat parvovirus 1 (MG693107.1) [8], and *E*. *helvum* bat parvovirus 2 (JX885610) [9], *Desmodus rotundus* bat parvovirus (NC032097.1) [10], simian parvo-like virus 3 (KT961660.1) [11], Turkey parvovirus TP1-2012/Hun (KF925531) [12], porcine parvovirus 7 (KU563733) [13], murine chapparvovirus (MF175078) [14], Tasmanian devil-associated chapparvovirus strains 1–6 (MK513528-MK53533) [15], red-crowned crane-associated parvovirus (KY312548, KY312549, KY312550, KY312551) [16], and chicken chapparvovirus 1 and 2 (MG846441 and MG846642) [17]. A close relative of murine chapparvovirus, initially reported in the feces of a wild *Mus musculus* from New York City [14], called murine kidney parvovirus (MH670588) was recently shown to cause nephropathy in immunocompromised laboratory mice [18]. A recent survey of eukaryotic genomes for chapparvovirus sequences has also shown the presence of a likely exogeneous chapparvovirus genome in a fish (Gulf pipefish or *Syngnathus scovelli*) and of mostly defective germline sequences in another fish (Tiger tail seahorse or *Hippocampus comes*) as well as in multiple invertebrates, indicating an ancient origin for chapparvoviruses [19]. A phylogenetic analysis of NS1 also indicated chapparvoviruses fall outside the traditional vertebrate-infecting *Parvovirinae* subfamily clade and closer to that of a subset of members of the subfamily *Densovirinae* [19]. 

Here an unexplained diarrhea outbreak among dogs was analyzed using viral metagenomics after diagnostic tests were negative for common canine enteric pathogens. The genome of a novel chapparvovirus was characterized and used to perform an epidemiological study to measure its prevalence and possible clinical significance.

## 2. Materials and Methods

### 2.1. Sample Collection and Pathogen Screening

Nine stool samples from dogs suffering from an infectious diarrhea outbreak in Colorado in October 2017 were submitted to IDEXX Reference Laboratories, Inc. (Sacramento, CA, USA) for pathogen testing. Fourteen dogs were involved in the initial outbreak which were identified by clinical signs that started with steatorrhea, progressed to hemorrhagic diarrhea with additional symptoms of lethargy, fever, and low lymphocyte counts pointing to a possible viral infection. At the time of feces collection, the nine sampled dogs were at various stages of the disease, with two of the dogs relapsing a month after initially experiencing parvo-like clinical signs. These stool samples were all negative for *Giardia* spp., *Cryptosporidium* spp., *Salmonella* spp., *Clostridium perfringens* enterotoxin gene (quantitative), *Clostridium perfringens* Alpha-toxin gene (quantitative), Canine enteric coronavirus (alphacoronavirus), Canine Parvovirus 2 and Canine Distemper virus using the IDEXX canine diarrhea profile real-time PCR tests.

### 2.2. Metagenomic Analysis

Stool samples were grouped into three pools of three and vortexed in phosphate buffer saline (PBS) with zirconia beads followed by microfuge centrifugation at 14,000 *rpm* for 10 min. The supernatants were passed through a 0.45 µm filter (Millipore, Burlington, MA, USA) and digested with a mixture of nuclease enzymes to enrich for viral particles [20,21]. RNA was extracted using the MagMAX kit (ThermoFisher, Waltham, MA, USA) which was transcribed into cDNA using a random RT-PCR step. The library was generated using the transposon-based Nextera™ XT Sample Preparation Kit (Illumina, San Diego, CA, USA) which was deep sequenced with the MiSeq platform (250 bases, paired-end reads) with dual barcoding. After demultiplexing the reads, they were trimmed and de novo assembled to produce contigs [22]. Both singlets and contigs were compared to all eukaryotic viral protein sequences in GenBank’s non-redundant database using BLASTx [23]. 

### 2.3. Genome Assembly and Diagnostic PCR

Pairwise identity matrices using the amino acid sequence of the NS1 wasgenerated using Geneious R11 (Newark, NJ, USA). Amino acid sequences from the NS1 region of all available chapparvoviruses were aligned using MUSCLE and a Maximum likelihood tree was created using the Jones–Taylor–Thorton matrix-based model with 1,000 bootstrap replicates in MEGA6.0 [24,25,26].

A set of nested PCR primers were designed to screen for cachavirus in the nine stool samples from the diarrheal outbreak. DNA was extracted from each individual stool sample using the QIAamp MinElute Virus Spin kit (Qiagen, Hilden, Germany) and nested PCR assay primers were used to screen for cachavirus DNA. The first round of primers CPV_625F (5’-CAA CTA GCC GAA TGC AGG GA-3’) and CPV_948R (5’-CGA TAA CAT CCC CGG ACT GG-3’) were designed to target 323 nt of the NS1 region. The second round of primers CPV18_687FN (5’-AGC TCA GTT TGG CCC AGA TC-3’) and CPV_911RN (5’-AGAGGGATCGCTGGATCTGT-3’) targeted a 224 nt region within the amplicon of the first round of primers. The PCR (containing a final concentration of 0.2 µm of each primer, 0.2 mM of dNTPs, 0.625 U of Amplitaq Gold^®^ DNA polymerase (Applied Biosystems, Waltham, MA, USA), 1× PCR Gold buffer II, 1.5 mM of MgCl_2_ and 1 µL of DNA template in a 25 µl reaction) proceeded as follows: 95 °C for 5 min, 40 cycles of (95 °C for 30 s, (52 °C for the first round and 54 °C for the second round of primers) for 30 s, and 72 °C for 30 s), followed by a final extension at 72 °C for 7 min. PCR products of the correct size were verified by gel electrophoresis and Sanger sequencing. 

### 2.4. Prevalence 

A proprietary real-time PCR assay with an amplification efficiency of 95% and an r^2^ value of 0.99 was developed by IDEXX. One gram of feces was added to 3 mL of lysis buffer and 600 µL extracted into 200 µL nucleic acid eluate. Five µL of the eluate was tested in a PCR reaction with a limit of detection of 10 copies DNA for a sensitivity of 1,600 copies per gram of feces. A chi-square test comparing the proportion of healthy dogs that tested positive for cachavirus and those testing positive with diarrhea in 2017 and 2018 was performed in order to determine if the difference in frequency was statistically significant. 

## 3. Results

Nine canine diarrheal samples from an unexplained outbreak of diarrhea were analyzed by viral metagenomics using three pools of three diarrhea samples each. Based on the BLASTx results, one of the three pools showed the presence of viral sequences most closely related to different chapparvoviruses reported from different vertebrates (0.05% of all reads). Other eukaryotic viral sequences observed were from Gyrovirus 4 (0.0003% of all reads), which has been reported in both chicken meat and human stool [27], indicating that it likely represents a dietary contaminant, and Torque teno canis virus (0.002% of reads), a common commensal canine blood virus [28]. 

**Figure 1:**
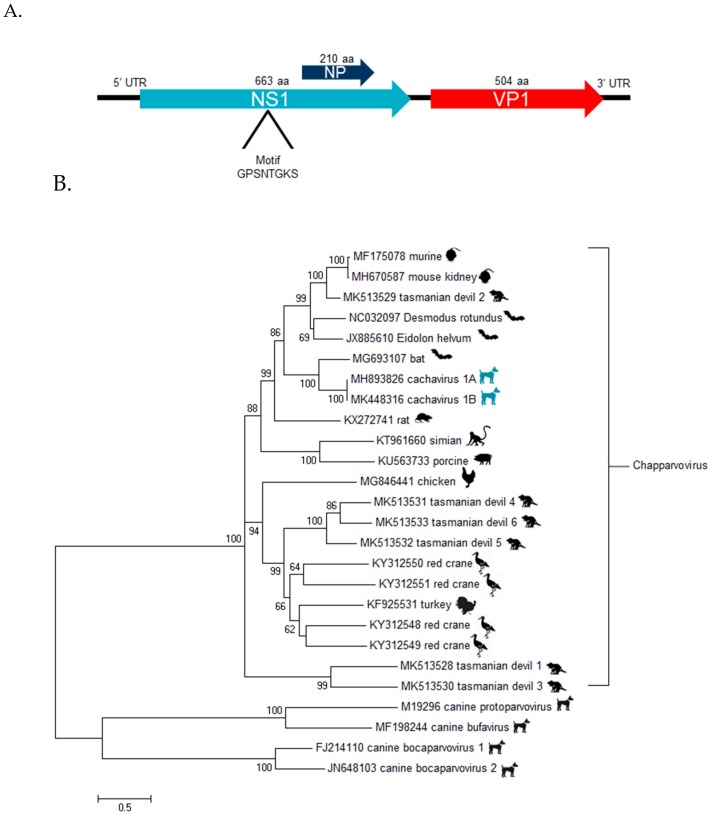
(**A**) ORF map and location of nucleotide-binding Walker loop motif. (B) Maximum likelihood tree of NS1 aa sequences of chapparvoviruses. Bar, 0.5 amino acid substitutions per site. Bootstrap values below 60 were removed.

Using de novo assembly and PCR paired with Sanger sequencing, a near complete genome of 4,123 bases containing the two main open reading frames of chapparvoviruses was generated (Figure 1, panel A). The available genome consisted of a 516 bases partial 5’UTR followed by an ORF encoding a 663 aa non-structural protein (NS) possessing the ATP binding Walker loop motif GPSNTGKS followed by a second ORF encoding a 505 aa viral capsid (VP) finishing with a 108 bases partial 3’UTR (Figure 1, panel A). When NS1 and VP1 proteins were compared to all available parvovirus sequences, the closest relative was from a Cameroonian fruit bat chapparvovirus (MG693107.1) [8] with an amino acid identity of 61 and 63% respectively (Table S1). A 210 amino acid ORF that is missing a start codon and is overlapping the NS1 ORF was also detected showing 57% identity to its homologue protein in mouse kidney parvovirus (AXX39021) [18] (Figure 1, panel A). This NP ORF is widely conserved among chapparvoviruses [19]. The 5’ UTR DNA sequence was 68% identical to that of the bat parvovirus sequence (MG693107.1)). The virus was named cachavirus (canine chapparvovirus) strain 1A (CachaV-1A).

Distance matrices of the NS1 showed that the cachavirus is sufficiently divergent based on ICTV criteria [6] (members of same species showing >85% NS1 identity) to qualify as a member of a tentative new species *Carnivore chapparvovirus species* 1 in the proposed *Chapparvovirus* genus (Table S1). A phylogenetic analysis of the NS1 ORF confirms its closest currently known relative is from a Cameroonian fruit bat (Figure 1, panel B). 

Using a nested PCR, the other 8 samples were tested for the presence of this virus which was detected in a second diarrheic sample from that outbreak. 

A larger set of canine fecal samples were then tested using a real-time PCR assay. Of 2,053 fecal samples tested, a total of 80 were positive (Table 1). Fecal sample submissions from the same time frame as the outbreak (Sept-Oct 2017) were tested in order to determine the prevalence of CachaV-1 during that time. Healthy samples from fecal flotation samples submitted in 2018 for preventive care screening were available. A second set of diarrhea samples that were collected during the same time frame as the healthy samples was also analyzed to check for differences in prevalence across time, as was a set of 83 bloody diarrhea samples. 

Three stool samples out of 203 healthy animals tested positive, 32 were positive out of 802 diarrhea submissions from September to October of 2017, and 45 were positive out of 965 diarrhea submissions from September to October of 2018. None of the 83 bloody diarrhea samples tested were positive (Table 1). When the fraction of PCR positive fecal samples was compared between the healthy animals (1.47% positive) and those with diarrhea, a statistically significant difference (*p* < 0.05) could be detected with the 965 diarrhea cases from 2018 (4.66% positive; *p* = 0.037), but not with the 803 diarrhea cases collected in 2017 (4.0% positive; *p* = 0.08). When 2017 and 2018 diarrhea samples were combined (4.35% positive) and compared to the healthy group (1.47% positive), we measured a *p*-value of 0.05. 

Cachavirus viral load as reflected by the Ct value of the real-time PCR were low across all four cohorts, with Ct values ranging from 29 to 39 with an average value of 36 for all positive groups. The five dog samples with the lowest Ct values (highest viral load) were then analyzed by viral metagenomics. All five samples yielded cachavirus reads, but one yielded a near complete genome (cachavirus [1B]). This sample also yielded 0.001% reads that were related to anelloviruses. None of the other four animals showed the presence of other known mammalian viruses. The cachavirus-1B genome showed 98% overall nucleotide identity with the index IDEXX-1A strain. The NS1 and VP encoded protein showed 99 % identity. 

## 4. Discussion 

There are currently five other known canine parvovirus species belonging to two genera of the *Parvoviridae* family. Canine parvovirus 2 (CPV2) in the *Carnivore protoparvovirus 1* species is a highly pathogenic virus that is closely related to feline parvovirus (FPV), the cause of feline panleukopenia, and can infect other carnivores such as coyotes, wolfs, raccoons and pumas [29]. Canine bufavirus, a second protoparvovirus (in the species *Carnivore protoparvovirus 2*) was reported in 2018 in fecal and respiratory samples from both healthy and dogs with signs of respiratory illness [30]. That same protoparvovirus was recently reported as a frequent component of juvenile cats fecal and respiratory samples [31]. The canine minute virus (CnMV) in the *Carnivore bocaparvovirus 1* species is less pathogenic than CPV2 but can cause diarrhea in young pups and is frequently found in the context of co-infections [32]. Distantly related to CnMV, a second canine bocavirus in the *Carnivore bocaparvovirus 2* species was sequenced in dogs with respiratory diseases [33]. A third bocavirus was then characterized from the liver of a dog with severe hemorrhagic gastroenteritis [34]. 

Here, we describe the near complete genomes of two closely related cachaviruses, members of a new tentative species (*Carnivore chapparvovirus 1*) in a proposed genus *Chapparvovirus*, the third genera of viruses from the *Parvoviridae* family now reported in canine samples. The chapparvovirus was found in only two animals of the initial nine sampled. Many of the dogs in the outbreak analyzed were sampled more than 10 days after onset of clinical signs, increasing the possibility that they were no longer shedding viruses. Additionally, diarrhea is one of the top reasons for veterinary visits and some patients may have coincidentally presented with diarrhea from some other cause. 

The two samples positive for CachaV-1 presented in the same week and were in the group of patients with the most severe clinical signs, requiring plasma transfusion and more aggressive supportive care. One of the two dogs, sampled at nine days after onset, died two days later. Because of the variable and often delayed feces sampling, it was therefore not possible to determine a clear disease association in this small group of diarrheic dogs (i.e., not all affected animals were shedding cachavirus). 

A possible role for the cachavirus infection in canine diarrhea was further tested by comparing cachavirus DNA PCR detection in larger groups of healthy and diarrheic animals including a group of animals with bloody diarrhea. A statistically significant difference (*p* = 0.037) was seen when diarrhea samples from 2018 were compared to the feces from healthy animals collected the same year. When 2017 diarrheic samples were compared to e 2018 healthy samples, the *p*-value was 0.08. When 2017 and 2018 diarrhea samples were combined and compared to the healthy samples, the *p*-value was 0.05. The association of cachavirus with diarrhea is therefore borderline and the detection of viral DNA remains limited to ~4% of cases of diarrhea. The limited number of healthy samples available for PCR limited the statistical power of this analysis and a larger sample size will be required for further testing of disease association. The absence of detectable cachavirus DNA in 83 other cases of bloody diarrhea was unexpected given the similar signs that developed in the initial outbreak. Detection of viral DNA in feces may be related to timing of sample collection as shedding of the intestinal lining during hemorrhagic diarrhea may preclude viral replication and fecal shedding. 

The detection of this virus in multiple fecal samples, the absence of prior cachavirus reports from tissues or fecal samples from other animals, and the confirmed vertebrate (murine) tropism of another chapparvovirus (mouse kidney parvovirus) [18], support the tentative conclusion that cachavirus infects dogs. Given its relatively low viral load and only borderline association with diarrhea, this virus’ possible role in canine diarrhea or other diseases will require further epidemiological studies. Because viral nucleic acids in fecal samples may also originate from ingestion of contaminated food (rather than replication in gut tissues), the tropism of cachavirus for dogs will require further confirmation such as specific antibody detection, viral culture in canine cells, and/or evidence of replication in vivo such as RNA expression in enteric tissues of dogs shedding cachavirus DNA. 

## Figures and Tables

**Table 1:** Real-time PCR results of cachavirus from four cohorts. A total of 2,053 samples were tested.

	Healthy Stool	Bloody Diarrhea	Diarrhea Submissions Sept-Oct 2017	Diarrhea Submissions Sept-Oct 2018	Total Diarrhea submissions (2017 + 2018)
number	203	83	802	965	1767
# Tested positive	3	0	32	45	77
Frequency	1.47%	0%	3.99%	4.66%	4.35%
Average Ct	36.49	-	36.48	36.38	36.15
*p*-value of frequency when compared to healthy cohort	-	-	0.08	0.037	0.05

## Supplementary Materials

The following are available online at https://www.mdpi.com/1999-4915/11/5/398/s1, Table S1: Percent identity between NS1 proteins of chapparvoviruses

## References

[1] Cave N.J., Marks S.L., Kass P.H., Melli A.C., Brophy M.A. (2002). Evaluation of a routine diagnostic fecal panel for dogs with diarrhea. J. Am. Vet. Med. Assoc..

[2] Nandi S., Kumar M. (2010). Canine parvovirus: Current perspective. Indian J. Virol..

[3] Conteville L.C., Zanella L., Marín M.A., Filippis A.M.B.d., Nogueira R.M.R., Vicente A.C.P., Mendonça M.C.L.d. (2015). Parvovirus B19 1A complete genome from a fatal case in Brazil. Mem. Inst. Oswaldo Cruz.

[4] Pérez R., Calleros L., Marandino A., Sarute N., Iraola G., Grecco S., Blanc H., Vignuzzi M., Isakov O., Shomron N. (2014). Phylogenetic and genome-wide deep-sequencing analyses of canine parvovirus reveal co-infection with field variants and emergence of a recent recombinant strain. PLOS ONE.

[5] Miranda C., Parrish C.R., Thompson G. (2014). Canine parvovirus 2c infection in a cat with severe clinical disease. J. Vet. Diagn. Invest..

[6] Cotmore S.F., Agbandje-McKenna M., Canuti M., Chiorini J.A., Eis-Hubinger A.M., Hughes J., Mietzsch M., Modha S., Ogliastro M., Penzes J.J. (2019). ICTV Virus Taxonomy Profile: Parvoviridae. J. Gen. Virol..

[7] Yang S., Liu Z., Wang Y., Li W., Fu X., Lin Y., Shen Q., Wang X., Wang H., Zhang W. (2016). A novel rodent Chapparvovirus in feces of wild rats. Virol. J..

[8] Yinda C.K., Ghogomu S.M., Conceicao-Neto N., Beller L., Deboutte W., Vanhulle E., Maes P., Van Ranst M., Matthijnssens J. (2018). Cameroonian fruit bats harbor divergent viruses, including rotavirus H, bastroviruses, and picobirnaviruses using an alternative genetic code. Virus Evol..

[9] Baker K.S., Leggett R.M., Bexfield N.H., Alston M., Daly G., Todd S., Tachedjian M., Holmes C.E., Crameri S., Wang L.F. (2013). Metagenomic study of the viruses of African straw-coloured fruit bats: Detection of a chiropteran poxvirus and isolation of a novel adenovirus. Virology.

[10] Souza W.M., Romeiro M.F., Fumagalli M.J., Modha S., de Araujo J., Queiroz L.H., Durigon E.L., Figueiredo L.T., Murcia P.R., Gifford R.J. (2017). Chapparvoviruses occur in at least three vertebrate classes and have a broad biogeographic distribution. J. Gen. Virol..

[11] Kapusinszky B., Ardeshir A., Mulvaney U., Deng X., Delwart E. (2017). Case-Control Comparison of Enteric Viromes in Captive Rhesus Macaques with Acute or Idiopathic Chronic Diarrhea. J. Virol..

[12] Reuter G., Boros A., Delwart E., Pankovics P. (2014). Novel circular single-stranded DNA virus from turkey faeces. Arch. Virol..

[13] Palinski R.M., Mitra N., Hause B.M. (2016). Discovery of a novel Parvovirinae virus, porcine parvovirus 7, by metagenomic sequencing of porcine rectal swabs. Virus Genes.

[14] Williams S.H., Che X., Garcia J.A., Klena J.D., Lee B., Muller D., Ulrich W., Corrigan R.M., Nichol S., Jain K. (2018). Viral Diversity of House Mice in New York City. MBio.

[15] Chong R., Shi M., Grueber C.E., Holmes E.C., Hogg C., Belov K., Barrs V.R. (2019). Fecal viral diversity of captive and wild Tasmanian devils characterized using virion-enriched metagenomics and meta-transcriptomics. J. Virol..

[16] Wang Y., Yang S., Liu D., Zhou C., Li W., Lin Y., Wang X., Shen Q., Wang H., Li C. (2019). The fecal virome of red-crowned cranes. Arch. Virol..

[17] Lima D.A., Cibulski S.P., Tochetto C., Varela A.P.M., Finkler F., Teixeira T.F., Loiko M.R., Cerva C., Junqueira D.M., Mayer F.Q. (2019). The intestinal virome of malabsorption syndrome-affected and unaffected broilers through shotgun metagenomics. Virus Res..

[18] Roediger B., Lee Q., Tikoo S., Cobbin J.C.A., Henderson J.M., Jormakka M., O’Rourke M.B., Padula M.P., Pinello N., Henry M. (2018). An Atypical Parvovirus Drives Chronic Tubulointerstitial Nephropathy and Kidney Fibrosis. Cell.

[19] Pénzes J.J., de Souza W.M., Agbandje-McKenna M., Gifford R.J. (2019). An ancient lineage of highly divergent parvoviruses infects both vertebrate and invertebrate hosts. bioRxiv.

[20] Allander T., Emerson S.U., Engle R.E., Purcell R.H., Bukh J. (2001). A virus discovery method incorporating DNase treatment and its application to the identification of two bovine parvovirus species. Proc. Nat. Acad. Sci. USA.

[21] Victoria J.G., Kapoor A., Li L., Blinkova O., Slikas B., Wang C., Naeem A., Zaidi S., Delwart E. (2009). Metagenomic Analyses of Viruses in Stool Samples from Children with Acute Flaccid Paralysis. J. Virol..

[22] Deng X., Naccache S.N., Ng T., Federman S., Li L., Chiu C.Y., Delwart E.L. (2015). An ensemble strategy that significantly improves de novo assembly of microbial genomes from metagenomic next-generation sequencing data. Nucleic Acids Res..

[23] Zaretskaya I., Johnson M., McGinnis S., Raytselis Y., Merezhuk Y., Madden T.L. (2008). NCBI BLAST: a better web interface. Nucleic acids research.

[24] Tamura K., Stecher G., Peterson D., Filipski A., Kumar S. (2013). MEGA6: Molecular Evolutionary Genetics Analysis version 6.0. Mol. Boil. Evol..

[25] Edgar R.C. (2004). MUSCLE: Multiple sequence alignment with high accuracy and high throughput. Nucleic Acids Res..

[26] Jones D.T., Taylor W.R., Thornton J.M. (1992). The rapid generation of mutation data matrices from protein sequences. Comput. Appl. Biosci..

[27] Chu D.K., Poon L.L., Chiu S.S., Chan K.H., Ng E.M., Bauer I., Cheung T.K., Ng I.H., Guan Y., Wang D. (2012). Characterization of a novel gyrovirus in human stool and chicken meat. J. Clin. Virol..

[28] Okamoto H., Takahashi M., Nishizawa T., Tawara A., Fukai K., Muramatsu U., Naito Y., Yoshikawa A. (2002). Genomic characterization of TT viruses (TTVs) in pigs, cats and dogs and their relatedness with species-specific TTVs in primates and tupaias. J. Gen. Virol..

[29] Allison A.B., Kohler D.J., Fox K.A., Brown J.D., Gerhold R.W., Shearn-Bochsler V.I., Dubovi E.J., Parrish C.R., Holmes E.C. (2013). Frequent cross-species transmission of parvoviruses among diverse carnivore hosts. J. Virol..

[30] Vito M., Gianvito L., Eszter M.K., Szilvia M., Renáta V.-K., Eszter K., Barbara Di M., Michele C., Nicola D., Canio B. (2018). Novel Parvovirus Related to Primate Bufaviruses in Dogs. Emerg. Infect. Dis. J..

[31] Diakoudi G., Lanave G., Capozza P., Di Profio F., Melegari I., Di Martino B., Pennisi M.G., Elia G., Cavalli A., Tempesta M. (2019). Identification of a novel parvovirus in domestic cats. Vet. Microbiol..

[32] Carmichael L.E., Schlafer D.H., Hashimoto A. (1991). Pathogenicity of minute virus of canines (MVC) for the canine fetus. Cornell Vet..

[33] Kapoor A., Mehta N., Dubovi E.J., Simmonds P., Govindasamy L., Medina J.L., Street C., Shields S., Lipkin W.I. (2012). Characterization of novel canine bocaviruses and their association with respiratory disease. J. Gen. Virol..

[34] Li L., Pesavento P.A., Leutenegger C.M., Estrada M., Coffey L.L., Naccache S.N., Samayoa E., Chiu C., Qiu J., Wang C. (2013). A novel bocavirus in canine liver. Virol. J..

